# Selective Laser Melting of Ti-6Al-4V: The Impact of Post-processing on the Tensile, Fatigue and Biological Properties for Medical Implant Applications

**DOI:** 10.3390/ma13122813

**Published:** 2020-06-22

**Authors:** Parastoo Jamshidi, Miren Aristizabal, Weihuan Kong, Victor Villapun, Sophie C. Cox, Liam M. Grover, Moataz M. Attallah

**Affiliations:** 1 School of Materials and Metallurgy, University of Birmingham, Edgbaston B15 2TT, UK; p.jamshidi@bham.ac.uk (P.J.); WXK744@student.bham.ac.uk (W.K.); 2 Ceit, Manuel Lardizabal 15, 20018 Donostia/San Sebastián, Spain; maristizabal@ceit.es; 3 Tecnun School of Engineering, Universidad de Navarra, Manuel Lardizabal 13, 20018 Donostia/San Sebastián, Spain; 4 School of Chemical Engineering, University of Birmingham, Edgbaston B15 2TT, UK; V.M.Villapun@bham.ac.uk (V.V.); s.c.cox@bham.ac.uk (S.C.C.); L.M.Grover@bham.ac.uk (L.M.G.)

**Keywords:** selective laser melting, Post-processing, titanium alloys, mechanical properties

## Abstract

One of the main challenges in additive manufacturing (AM) of medical implants for the treatment of bone tissue defects is to optimise the mechanical and biological performance. The use of post-processing can be a necessity to improve the physical properties of customised AM processed implants. In this study, Ti-6Al-4V coupons were manufactured using selective laser melting (SLM) in two build orientations (vertical and horizontal) and subsequently post-processed using combinations of hot isostatic pressing (HIP), sandblasting (SB), polishing (PL) and chemical etching (CE). The effect of the different post-manufacturing strategies on the tensile and fatigue performance of the SLMed parts was investigated and rationalised by observing the surface topography. Vertically built samples showed higher yield strength (YS) and ultimate tensile strength (UTS) than the horizontal samples, increasing from 760.9 ± 22.3 MPa and 961.3 ± 50.2 MPa in the horizontal condition to 820.09 ± 16.5 MPa and 1006.7 ± 6.3 MPa in the vertical condition, respectively. After the HIP treatment, the ductility was substantially improved in both orientations; by 2.1 and 2.9 folds in the vertical and horizontal orientations, respectively. The vertically built samples demonstrated a superior ductility of 22% following HIP and polishing. Furthermore, chemical etching was found to be the most effective surface post-processing treatment to improve the fatigue performance after HIP, achieving the highest run-out strength of 450 MPa. Most importantly, chemical etching after HIP enhanced the cellular affinity of the surface, in addition to its good fatigue performance, making it a promising post-processing approach for bone implants where tissue integration is needed.

## 1. Introduction

The development of additive manufacturing (AM) techniques has recently gained significant attention for the fabrication of customised implants to the needs of individual patients in healthcare [1,2]. This strategy relies on 3D printing of the part from digital models in a layer-by-layer fashion in an attempt to add new implant functionality with better specifications in less time and at a lower cost. Several investigations are being performed to evaluate the capacity of these techniques in comparison with the conventional routes [3,4,5]. Selective laser melting (SLM), one of the most common AM techniques, has been recently used in various studies to exploit the design flexibility of this technique in manufacturing patient-particular metallic implant parts. This system uses a high power density laser to melt and fuse the metallic powders according to the CAD model of the implant to form a 3D part in an inert atmosphere preventing the oxidation of the metallic powder in the air [6]. Besides, SLM, similar to other AM techniques, can reduce the amount of materials waste, in addition to its disruptive benefits of saving time and cost by skipping many additional manufacturing steps to obtain the final part [7]. Despite the novelty of this technology, there are still key knowledge gaps for future research to improve the capability of the AM implant for clinical application. The microstructure, surface topography and mechanical properties of SLM-fabricated implants are the key features that need to be assessed for their reliable clinical use [1]. The biological performance of AM metallic implants and the interaction between the implant and the surrounding tissue are all dependent on the quality of these features. Thus, controlling the various AM process parameters during the build, part cleaning, finishing after the build and the use of several post-processing steps are required to improve the surface topography, mechanical properties and biological performance in their application [2]. Using the design flexibility of AM, the mechanical properties, implant design (e.g., the use of lattices and engineered porosity) and implant surface finish can be tailored to minimise stress shielding and maximise the biological response, respectively. Recently, Cox et al. demonstrated that the surface finishing of SLMed Ti-6Al-4V parts significantly alters surface topography, resulting in a direct influence on the cellular and biofilm adhesion [3]. Hot isostatic pressing (HIP), polishing, sandblasting and chemical etching are among the post-processing methods that are routinely used as standard procedures to improve the mechanical properties and surface topography of SLMed components [4,5,6]. For example, HIP, in which the materials go through pressurised heat treatment, is reported by various studies to be a necessary step for SLMed Ti implants due to its positive impact on the mechanical properties [7]. HIP helps reduce the residual pores that formed during SLM due to lack of fusion, argon gas entrapment within the gas atomised particles, or evaporation/keyholing, resulting in an improvement in fatigue performance [8,9]. HIP was also shown to result in a better cellular attachment and proliferation of seeded preosteoblast cells onto the SLMed implant surface [3]. Sandblasting and polishing, however, were shown to reduce the rate of cellular activity [3], increasing the risk of implant infection due to adherence of bacteria onto the surfaces. As such, an appropriate surface finishing approach that promotes structural integrity while enabling mammalian cell adhesion and preventing bacterial colonisation is still required for customised medical devices. For most joint arthroplasty, the strength and fatigue performance of the implants are critical factors that need to meet specific criteria, especially for high load-bearing applications. Four important factors are responsible for fatigue crack initiation in parts made by AM; (i) internal defects, (ii) microstructure, (iii) surface condition and (iv) residual stresses. HIP can reduce the internal defects and residual stresses and modify the microstructure to a more fatigue-resistant microstructure. However, the surface condition remains as an important factor that needs to be addressed before the clinical application of AM to medical implants. 

Roudnicka et al. [4] demonstrated that the effect of the surface condition on the fatigue behaviour is more important than the influence of the internal defects, suggesting that the role of HIP on the fatigue performance of SLM Ti-6Al-4V alloy is secondary to the influence of surface treatment. Nonetheless, it appears that the post-processing methods such as HIP, machining and polishing are all essential steps to create AM implants with high structural integrity [10,11,12]. These post-processing treatments can significantly affect the mechanical and biological performance of the AM parts [3,13]. Despite the numerous studies on optimising the AM process parameters of metallic implants manufactured from stainless steels, CoCr alloys and Ti-alloys, fewer studies have evaluated the required post-processing steps that can achieve a balance between mechanical properties and biological performance of these components. In this study, Ti-6Al-4V coupons were manufactured using SLM in two build orientations (vertical and horizontal) and subsequently post-processed using different post-processing strategies to understand their impact on mechanical and biological performance. 

## 2. Method and Materials

### 2.1. Selective Laser Melting of Ti-6Al-4V Coupons and Mechanical Specimens 

**Figure 1:**
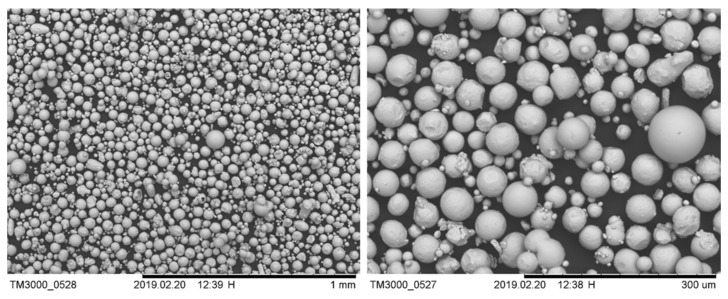
The morphology of the as-received Ti-6Al-4V gas atomised powder.

All the test specimens were fabricated using Ti-6Al-4V gas atomised powder (TLS Technik, Bitterfeld-Wolfen, Germany) size 20–50 μm on a Concept LaserM2 Cusing system (laser powder-bed fusion). The M2 system has a Yb-fibre laser with laser power up to 400 W and a 60 μm laser spot size, with the SLM process being carried out in an argon atmosphere, controlled to <100 ppm of O_2_. The samples were built using the following process parameters: 150 W laser power, 1750 mm/s scanning speed, 20 μm layer thickness and hatch spacing of 75 μm, based on the optimum parameters indicated in our previous studies [10,11]. The tensile and fatigue test pieces were built in two different orientations (vertical and horizontal) to study the anisotropic behaviour of the builds. Figure 1 shows the morphology of the powder used in this study. The morphology of the powder was spherical in shape, with limited satellites, suggesting a good flowability, which is essential to avoid recoating-induced defects. 

### 2.2. Post Processing of Additively Manufactured Parts 

Following SLM, HIPing was performed for all the samples using an EPSI HIP vessel (Temse, Belgium). Samples were HIPed at 930 °C/100 MPa/4 h, using a furnace heating/cooling rate of 5 °C/min to/from the dwell temperature.

Several surface treatment methods were then applied to assess their impact on the mechanical properties and biocompatibility. Sandblasting (SB) was used on a set of samples to improve the surface finish by removing the residual partially melted particle powder from the surface. SB is a standard procedure in industrial SLM operations, although its impact on biocompatibility is rarely assessed. SB was done in an air blast cabinet (CBI Equipment Ltd., Lytchett Matravers, UK) using a blasting gun with a compressed air regulator set to 4 bar. CARBOREX micro grit black abrasive powder with a size of 53 μm was used (Washington Mill Electro Minerals, Ramsbottom, UK). The grit blasting was performed for 30 min, with a speed of 100 m/s. A wet polishing (PL) process was also performed on a different set using centrifugal disc finishing machine (Finishing Techniques Ltd., FINTEK, Ramsbottom, UK) to produce completely smooth mirror-like surfaces. The process consists of two stages; parts were immersed in a centrifugation tank in two separate media of ceramic balls and plastic grinding chips to deburr and polish the parts for 7 h. Finally, a chemical etching (CE) process was also used on a third set as a single technique after HIP for the elimination of defective surface layers and partially melted particles. The samples were immersed into an etching solution (HF:HNO_3_:H_2_O = 1:2:3) for about 10 min and then transferred into distilled water for about 5 min and rinsed with ethanol to complete the cleaning stage. 

### 2.3. Material Characterisation

To characterise the surface morphology, microstructure and fracture surface of the various conditions, a Scanning Electron Microscope (SEM; Quanta200, FEI, CZ, Hillsboro, OR, US) was used. Electron Backscatter Diffraction (EBSD) mapping was performed using a field emission gun (FEG) SEM (NNS450, FEI, Hillsboro, OR, US), equipped with a DigiView (EDAX, Mahwah, NJ, USA) detector to evaluate the microstructure of the SLMed Ti-6Al-4V samples before and after the HIP treatment. A step size of 1.5 μm was used to capture high-quality EBSD maps. Phase identification was also performed by X-ray diffraction using a Rigaku Smartlab (Tokyo, Japan) diffractometer, with a Cu Kα tube, using a 2θ range of 30–80°. Finally, to investigate the effect of chemical etching on the surface topography and roughness, a G5 infinite focus profilometer (Alicona, Kent, UK) was used with a 1.624 × 1.624 mm scan area. A 3D point cloud was generated for each surface, alongside SEM imaging. 

#### 2.3.1. Tensile Testing

**Figure 2:**
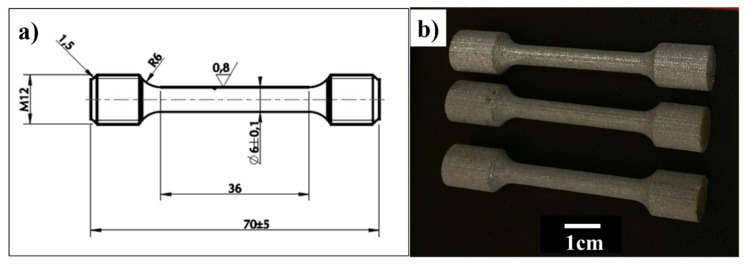
(**a**) ASTM E-8 tensile test geometry with d = 6 mm end sections for M12 threads machining, and (**b**) images of Ti-6Al-4V tensile test specimens manufactured via selective laser melting (SLM). Only the threads section was machined to examine the influence of post-processing on the gauge section.

Tensile testing was performed at room temperature using a servo-hydraulic Zwick/Roell (Ulm, Germany) tensile testing machine with an external extensometer on the SLMed parts for the different conditions. Cylindrical dog-bone-shaped specimens (according to ASTM E-8, Figure 2) were built using SLM in their shape in the vertical and horizontal orientations. Machining was only applied to the thread section, but the rest of the sample kept the as-fabricated surface, which was later post-processed using the various aforementioned methods. 

#### 2.3.2. Fatigue Testing

Specimens for fatigue test (10 × 10 × 80 mm^3^) were manufactured using SLM. Four types of samples were tested; as-fabricated, SLM + HIP, SLM + HIP + PL, and SLM + HIP + CE. Four-point bending fatigue tests were carried out using an Amsler Vibrophore electro-magnetic resonance testing machine for fatigue life evaluation. Tests were conducted per ASTM F1264 – 03 at room temperature using a stress ratio (R) of 0.1 and a frequency of ~70 Hz, with 1.0 × 10^7^ cycles being defined as the run-out life. The R ratio is the ratio of minimum stress to maximum stress (*σ*_min_/*σ*_max_) applied over the fatigue cycle. The relatively higher test frequency was used to speed up the test, keeping in mind that the test aimed to comparatively assess the fatigue behaviour of the different conditions, even if very limited thermal phenomena might be encountered. For each group, ~5 specimens were used for the test. The applied elastic maximum stress at the control surface *σ*_max_ was calculated using the elastic bending theory according to the following equation [12]:

(1) $$\sigma_{\max}=\frac{3P_{\max}\cdot S}{b\cdot t^{2}}$$

where *P*_max_ is the maximum applied load, *S* is the span between inner and outer rollers (20 mm), *b* is the specimen width and *t* is the specimen height. 

#### 2.3.3. In Vitro Testing

The biocompatibility of SLMed Ti-64 samples in the SLM + HIP condition and after CE was assessed using osteosarcoma human SAOS-2 cells, passage 18, previously grown at 37 °C (5% CO_2_, 95% air) in Dulbecco’s Modified Eagle’s Medium (DMEM, Sigma-Aldrich, St. Louis, MS, USA) supplemented with 10% foetal bovine serum (Sigma-Aldrich, St. Louis, MS, USA) and 1% penicillin/streptomycin (Sigma-Aldrich, St. Louis, MS, USA). The plastic coverslips were used as control samples. The samples were autoclaved, sterilised with 70% ethanol, and exposed overnight under ultraviolet light before in vitro experiments. Once previously grown SAOS-2 were semi confluent, cells were trypsinised, counted and diluted to a concentration of 400,000 cells/mL. After disinfection, samples were placed on 24 well plates containing 10mL of DPBS (Sigma-Aldrich, St. Louis, MS, USA) to prevent cell drying. Fifty microlitres (20,000 cells per sample) of the cell suspension was seeded onto the surfaces, spread using a pipette tip and left at room temperature for 15 min. Next, plates were moved into an incubator (37 °C, 5% CO_2_, 95% air) for 40 min and 1 mL of supplemented DMEM added. Media exchange was performed every three days with supplemented DMEM. After seeding, Alamar blue (Invitrogen, Renfrew, UK) was used as a noninvasive proliferation assay on days 3, 7 and 14. This assay is based on a redox indicator that yields a colourimetric change and fluorescent signal in response to metabolic activities. 1 mL of unmodified DMEM containing 10% AlamarBlue (Fisher Scientific Ltd., Loughborough, UK) was introduced in the seeded samples for each time point. After 4 h of incubation at 37 °C (5% CO_2_, 95% air), fluorescence measurements were carried out using a TECAN Spark plate reader (Tecan Trading AG, Männedorf, Switzerland) with an excitation and emission wavelengths of 560 nm and 590 nm, respectively. Before SEM imaging, incubated samples were collected at required time-points, and fixed in 1.5% glutaraldehyde (Sigma-Aldrich, Gillingham, UK) at 4 °C overnight. Cells were then dehydrated through a series of increasing concentrations of ethanol (50%, 70%, 80%, 90%, and 100%) for 10 min at each concentration. After dehydration, the cell-sample was critical-point-dried with CO_2_ and examined under SEM, as previously explained in Section 2.3.

## 3. Result and Discussion 

### 3.1. Microstructural Development

**Figure 3:**
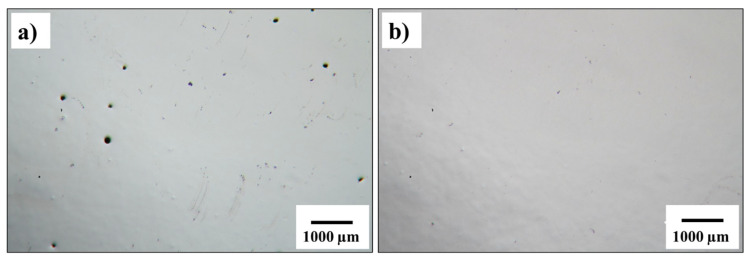
Porosity level in Ti-6Al-4V builds in the (**a**) as-fabricated condition showing spherical pores and (**b**) post-hot isostatic pressing (HIP) condition.

The effect of HIP on the density of SLMed Ti-6Al-4V was assessed to quantify its capability to eliminate the residual porosity within the matrix. The optical micrographs in Figure 3 show that the porosity level was significantly decreased after HIP (Figure 3b). HIP was effective in collapsing the internal porosity that may be caused by lack of fusion, entrapped argon in the feedstock powder during gas atomisation or from any stochastic gas entrapment during SLM. Entrapped gas pores result in near-spherical voids, within the solid matrix of the build [4], like those observed in the as-fabricated samples (Figure 3a). Application of HIP after SLM is necessary to reduce the presence of internal pores that are associated with AM, as the pores create stress concentration points and fatigue crack initiation sites [9,13,14]. It was previously reported that high-cycle fatigue life for the AMed bone implant can be improved using HIP [15].

**Figure 4:**
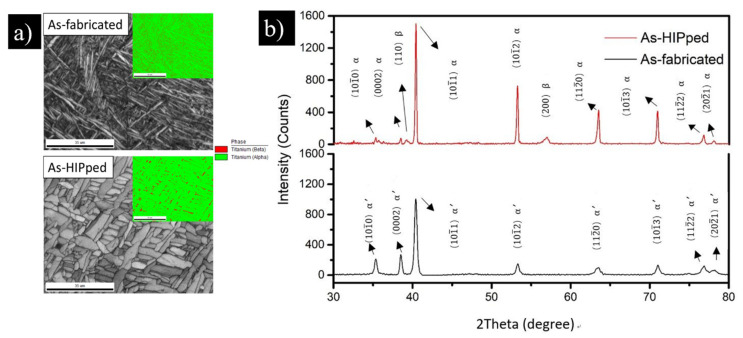
(**a**) EBSD phase map for as-fabricated and post-HIP Ti-64 parts and (**b**) XRD measurement of as-fabricated and post-HIP Ti-6Al-4V parts. Scale bars = 35µm.

The influence of HIP on the microstructural evolution was characterised using EBSD and XRD (Figure 4). The grain size and phase fractions changed after application of HIP, with the martensitic needle-like α′-structure in the as-fabricated Ti-6Al-4V transforming into α-plates and lamellae, with a small fraction of embedded β-Ti phase (Figure 4a), forming a basket weave microstructure. The microstructural change of SLMed Ti-6Al-4V from the martensitic structure into α + β microstructure following HIP has been previously reported in the literature to have a positive effect on the ductility [16]. The α-Ti (0002) and (10-10) peak intensities in the as-fabricated condition are significantly lower compared to the post-HIP Ti-6Al-4V condition (Figure 4b), whereas the β-peak became visible after HIP. The HIP process resulted in the coarsening of the microstructure, generating a coarse α-lamellar microstructure, with β between the lamellae.

### 3.2. Surface Finishing

**Figure 5:**
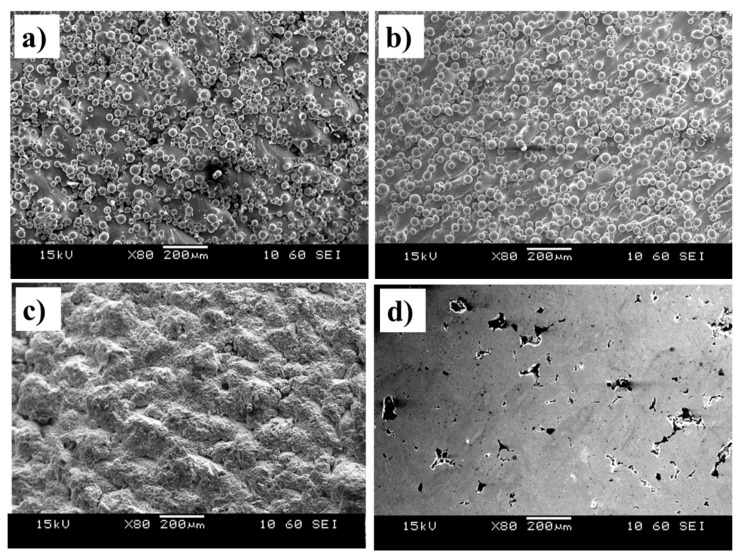
SEM images of top surfaces of (**a**) as-fabricated and samples with post-processing treatments; (**b**) HIP, (**c**) sandblasted and (**d**) polished SLMed Ti-6Al-4V parts.

Due to the inherent issues associated with the AM, as-fabricated parts normally exhibit partially melted particles on the surface, which can strongly affect both the tensile and fatigue properties [17]. To investigate the influence of the surface topography on tensile and fatigue performance of the SLMed parts, different post-processing treatments were performed. The surfaces of the samples after each post-processing treatment are illustrated in Figure 5. Partially melted particles were present on the surface topography of both the as-fabricated and post-HIP conditions (Figure 5a,b), with the only difference being in the less internal defects and porosity in the case of post-HIP samples (Figure 3b). 

The surface topography of the sandblasted and polished samples was altered by the removal of the partially melted particles (Figure 5c,d). No partially melted powder particles were observed after both sandblasting and polishing post-processing techniques. The sandblasted samples surface topography, however, contained troughs and peaks, which were significantly reduced in the case of the polished condition. Although polishing resulted in a mirror-finish, the process may struggle with the troughs and peaks associated with the subsurface partially melted particles or laser contouring defects (e.g., at the boundaries of the islands [16]), resulting in residual cavities similar to the ones shown in Figure 5d.

### 3.3. Tensile Properties

**Figure 6:**
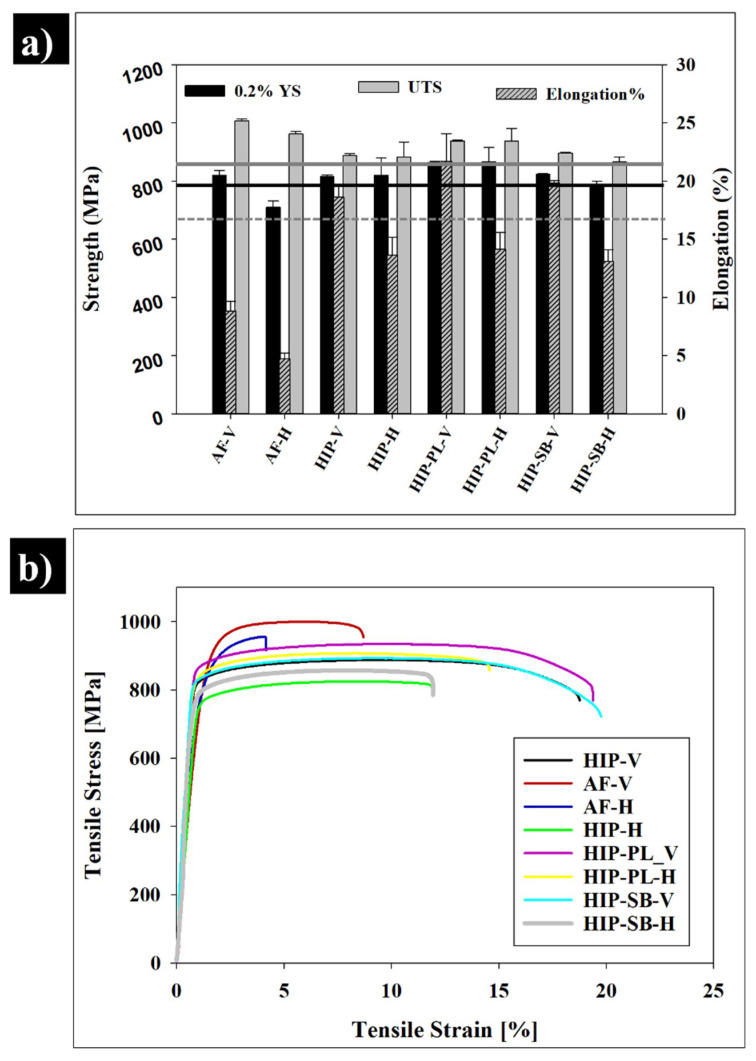
(**a**) Effect of post-processing on the tensile properties of SLMed Ti-6Al-4V. As-fabricated (AF), vertical orientation (V), horizontal orientation (H), hot-isostatic-pressed samples (HIP), polished samples (PL), and sandblasted samples (SB). (**b**) Typical tensile stress-strain curves for the as-fabricated condition and in various post-processing conditions.

The tensile properties of the Ti-6Al-4V samples built-in vertical and horizontal orientations at different conditions are shown in Figure 6a. There was a significant difference in the yield strength (YS, *p* < 0.05) and ultimate tensile strength (UTS, *p* < 0.005) between the vertical and horizontal orientations in the as-fabricated condition. Vertically built samples showed higher YS and UTS than the horizontal samples, increasing from 760.9 ± 22.3 MPa and 961.3 ± 50.2 MPa in the horizontal condition to 820.09 ± 16.5 MPa and 1006.7 ± 6.3 MPa in the vertical condition, respectively. This finding highlights the anisotropic behaviour of SLMed material, which is caused by the anisotropic microstructure, crystallographic orientation, and spatial distribution of defects concerning the build direction [16]. Earlier studies investigated the influence of the anisotropic microstructure caused by the build orientation on the tensile properties of the SLMed samples [18,19,20,21]. Qiu et al. showed that the SLMed Ti-6Al-4V built vertically (with columnar grain along the tensile axis) had higher YS and UTS compared with horizontality built samples, which was attributed to the orientation of the columnar grains perpendicular to the loading axis in the horizontal samples resulting in the anisotropy in mechanical properties. [16]. Figure 6b shows the engineering stress-strain plots obtained from selected tensile tests for both vertical and horizontal orientations after different post-processing conditions. The maximum elongation-to-failure was obtained in vertical orientation after the HIP + PL post-processed condition.

Following HIP, the UTS and YS slightly dropped due to the transformation of the fine α′ structure in the as-fabricated condition (Figure 4) into the α + β microstructure in both. However, the tensile properties obtained in this study for all post-processed conditions exceeded the minimum tensile properties in ASTM F3001-14 for AM of Ti-6Al-4V [22] (a minimum of 860 MPa for UTS, 795 MPa for YS, and 10% elongation), as shown in Figure 6. The tensile properties were also comparable to those reported in the literature and were generally consistent with the properties range for conventionally annealed Ti-6Al-4V in different conditions [20,23].

Generally, after HIP, the elongation is substantially increased in all the conditions and was a little higher than the ranges typically reported in the literature [23,24,25]. The tensile properties of the SLMed Ti-6Al-4V specimens produced in this study were compared to the wrought Ti-6Al-4V, wrought and annealed Ti-6Al-4V, as summarised in Table 1. The ductility was substantially improved in both orientation cases; vertically and horizontally by 2.1 and 2.9 folds, respectively. Many studies considered the strong influence of heat treatment and HIP on improving the ductility of SLMed Ti-6Al-4V [7,9,19]. Facchini et al. found out that the heat treatment modified and stabilised the microstructure by transforming the metastable martensite into a dual α + β microstructure. This microstructural development significantly enhanced the ductility up to 10.6 ± 0.6% [25]. Several other authors have shown that the residual stresses and the martensitic microstructure in the as-fabricated Ti-6Al-4V parts may result in lowered ductility. The formation of the β phase, even in limited volume fractions, can lead to an improvement in ductility [19]. Here it is demonstrated that the appearance of a small fraction of the β-Ti phase among the dominant large α-Ti plates after HIP (Figure 4b) had a positive effect on ductility (Figure 6). This appears to agree with the observation by other researchers [26,27].

In addition to phase composition and residual stress, the build orientation may affect the tensile properties, and in particular the ductility of the as-fabricated Ti-6Al-4V samples [7,19]. It was found in this study that the elongation in the vertical samples was higher than horizontally built samples (Figure 6). The HIP treatment was effective in increasing the elongation and ductility of the vertically built SLMed Ti-6Al-4V samples substantially, which is attributed to the combined effect of the collapse of the pores and the microstructural development associated with HIP. The superior ductility with elongation of ~22% was obtained for the samples built vertically and post-processed using HIP and polishing (HIP-PL-V). As a result, the HIP-PL samples were selected for further fractographic analysis. The fracture surface evaluation was performed on two different conditions of the as-fabricated and HIP-PL conditions at two different orientations to observe the influence of the post-processing route via HIP and polishing, as well as the build orientation.

**Figure 7:**
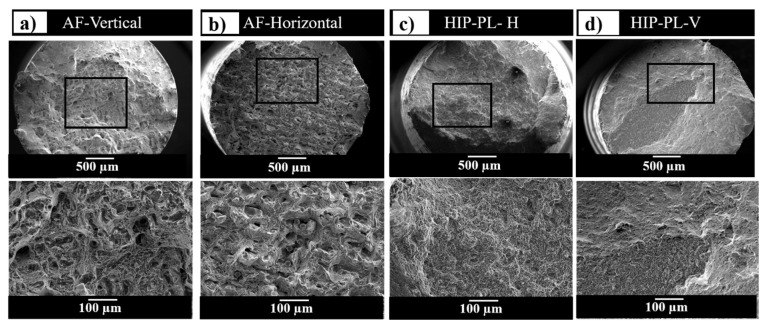
Tensile fracture surfaces of SLMed Ti-6Al-4V in two orientations and conditions; (**a**) as-fabricated (AF) vertical, (**b**) AF-horizontal, (**c**) hot-isostatic-pressed–polished (HIP + PL) horizontal, and (**d**) HIP + PL-vertical, showing an overall view of the fracture surface and magnified view of the boxed region below.

Selected fractographs for the various conditions are shown in Figure 7. The vertically built as-fabricated condition (Figure 7a) showed a rough and layered surface with the presence of open gas porosity and lack of fusion defects, from which the cracks initiated. The horizontally built samples (Figure 7b) exhibited a similar morphology, where the gas pores opened up during tensile loading. The presence of both large and small voids still can be observed macroscopically. These voids that form due to the stochastic nature of the process existed in multiple locations and acted as crack initiation sites. In these microstructures, the fracture is generally dominated by intergranular fracture mechanisms and, for this reason, the tensile ductility is reduced. There are still some differences in the layered morphology, which is due to the relative orientation of the prior-β grain boundaries to the loading direction [19]. Simonelli et al. previously assessed the influence of the crystallographic texture on the crack propagation in SLMed Ti-6Al-4V tensile test pieces at different orientations [19]. In the samples that were processed by HIP + PL, the fracture surface showed a much finer and more homogenous ductile dimple fracture morphology, compared with the as-fabricated condition. Notably, the presence of voids in these samples has been significantly reduced due to the impact of HIP, with the fracture surface becoming dominated by dimples [21].

**Figure 8:**
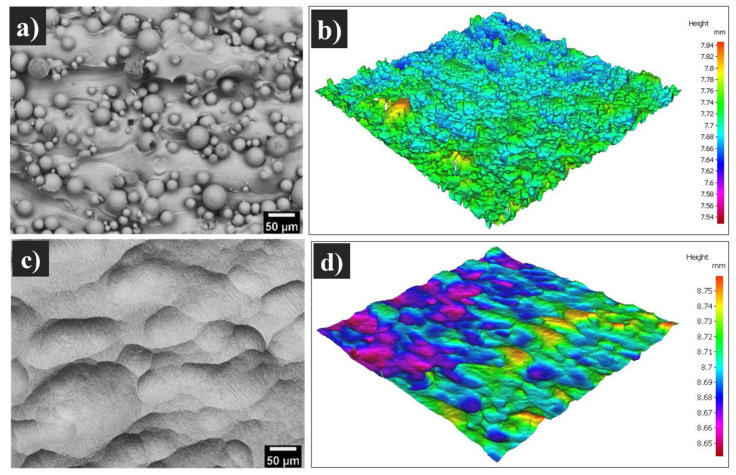
SEM micrographs and Alicona map of the top surfaces of (**a**,**b**) as-fabricated Ti-6Al-4V samples containing partially melted particles and (**c**,**d**) vertical condition after HIP + chemical etching.

Chemical etching as a surface treatment method for SLMed Ti-6Al-4V samples was further considered in this study to investigate the effect of this treatment on the fatigue behaviour of SLMed Ti-6Al-4V, compared with the other conditions. Based on our previous work [3], although polishing and sandblasting significantly alter the surface topography, they did not result in a favourable condition for cellular activity and caused a cytotoxic reaction by bacterial specimens adhesion onto these two types of surface finish. Thus, chemical etching, as a surface treatment method that is aimed towards improving the biocompatibility and mechanical properties for SLMed Ti-6AI-4V samples, was also investigated. Figure 8 shows the effect of chemical etching on the surface topography after HIP using the HF + HNO_3_ aqueous solution as a post-cleaning etchant. No remaining/partially melted particles can be observed on the surface of the SLMed parts after chemical etching (Figure 8c). This confirms the effectiveness of this method in reducing the roughness of the as-fabricated surface (Figure 8a), which has a direct impact on cellular adhesion and fatigue performance. A 3D reconstruction of the surface was obtained using the Alicona system, alongside the SEM surface micrograph. Surface roughness was significantly decreased after chemical etching (Figure 8d). This surface treatment led to a significant decrease in S_a_ from 12.2 ± 0.5 in the as-fabricated samples to 6.6 ± 0.3 µm after etching.

### 3.4. Fatigue Behaviour

**Figure 9:**
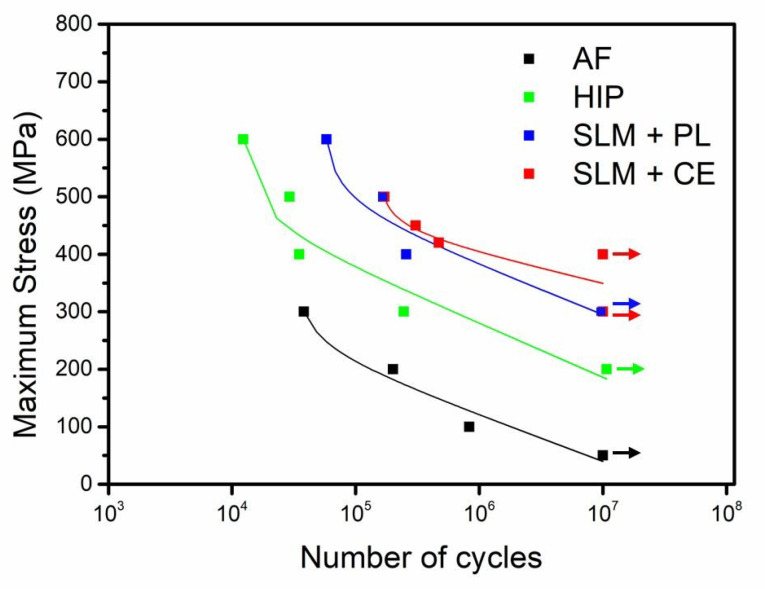
S-N curve of SLMed Ti-6Al-4V alloy in the form of as-fabricated (AF), compared with HIP, HIP and polishing (HIP + PL), and HIP and chemical etching (HIP + CE).

Fatigue testing was performed for the SLMed Ti-6Al-4V in four different conditions: as-fabricated, HIP only, HIP + polished, and HIP + chemically etched, as shown in Figure 9. The fatigue strength of the specimens was significantly improved after HIP, with significant improvement after polishing to mirror-finish and etching. Among the tested parts, the specimen post-processed by HIP + chemical etching exhibited the best fatigue performance, with a fatigue limit of ~450 MPa at 10^7^ cycles, which was higher than the other conditions, indicating the significant potential for chemical etching on improving the surface roughness and subsequently the fatigue behaviour. Immersion etching is a very simple and time-efficient procedure, compared with vibratory polishing. Chemical etching also has a potential for enhancing the cellular affinity of the surface while it holds high fatigue performance, compared with polishing which was previously shown to limit the cellular adhesion onto the polished surfaces.

**Figure 10:**
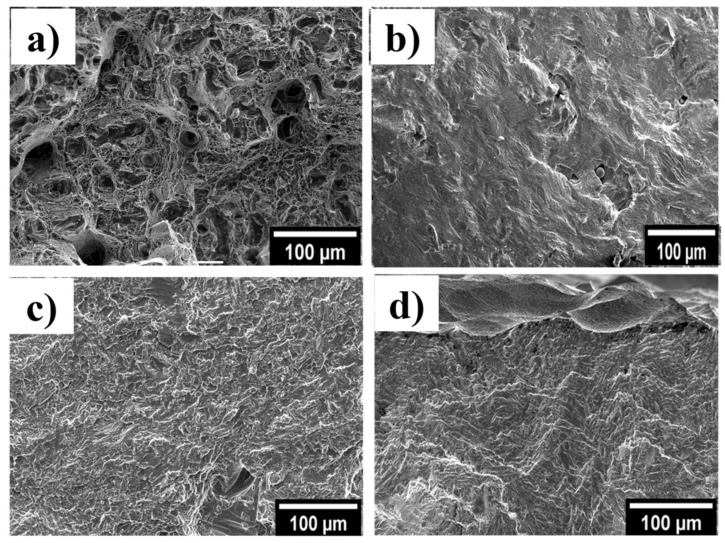
SEM images of fatigue fracture surfaces of (**a**) as-fabricated printed Ti-6Al-4V, (**b**) HIP only, (**c**) HIP + PL and (**d**) HIP + CE.

The fracture surface of fatigue test pieces revealed that the cracks originated from the partially melted particles on the surface in the as-fabricated condition leading to opened up gas pores and lack of fusion defects on the fracture surface (Figure 10a), which was exacerbated by the poor surface roughness. These features acted as fatigue crack initiation sites, resulting in low fatigue strength [28,29]. Multiple fracture initiation sites were observed, which led to the initiation of fracture [30]. The fatigue resistance of SLMed Ti-6Al-4V was improved following HIP, due to the residual stress relief and porosity reduction. The crack initiation in HIPed samples is attributed to the unconsolidated surface connected residual defects (Figure 10b), but the majority of the fracture surface demonstrated no opened up pores. Furthermore, the α + β microstructure of the HIP-treated condition also contributed to its highest fatigue strength, combined with the reduction of porosity and internal defects after HIP which act as crack initiation sites. In the case of the polished SLMed parts, the partially melted particles were also removed, resulting in higher fatigue strength in those samples. The fracture surface of samples that were processed by HIP + PL showed a much finer and more homogenous ductile dimple fracture morphology, compared with the as-fabricated condition (Figure 10c). The crack initiation site may be attributed to the dimpled intergranular facets in HIP + PL samples. The combination of HIP and chemical etching had a positive effect on the fatigue strength of the SLMed Ti-6Al-4V parts, achieving the highest run-out strength of 450 MPa (Figure 9). The fracture surface of HIP + Etching exhibited similar morphology to HIP + PL with finer and more homogenous ductile dimple fracture morphology (Figure 10d). This surface treatment etches out the crevices and irregularities from the surface, making it less susceptible to crack formation [29,31].

### 3.5. In Vitro Adhesion and Proliferation of Osteosarcoma Human SAOS-2 Cells on Chemical Etched Ti-6Al-4V Parts

**Figure 11:**
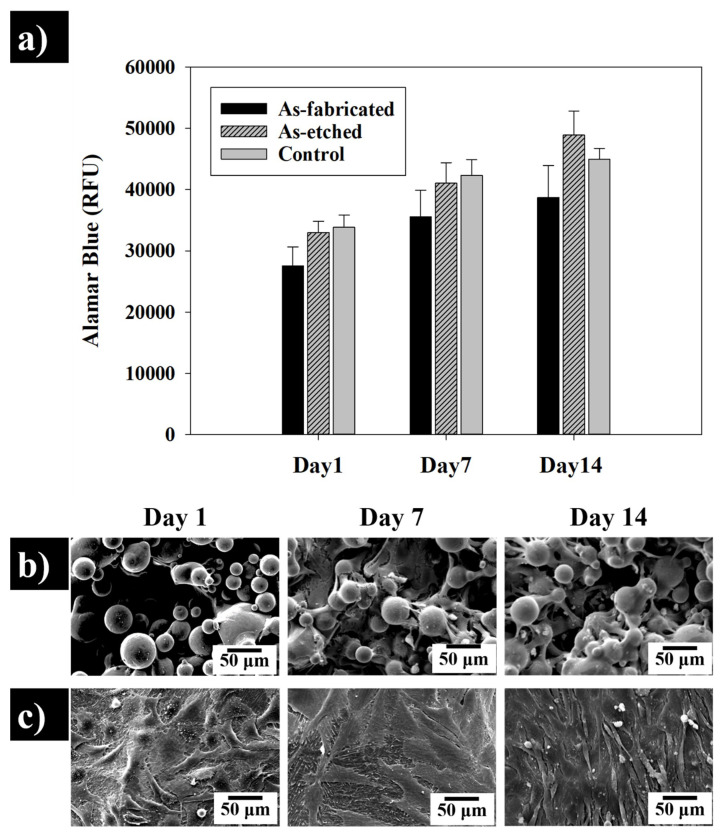
(**a**) Comparison of the metabolic activity of SAOS-2 cells seeded on as-fabricated Ti-6Al-4V surface, as-etched and control (tissue culture plastic) surface and (**b**,**c**) SEM micrographs of the seeded cells grown on the surface of SLMed Ti-6Al-4V in as-fabricated status and after etching on days 1, 7 and 14.

The adhesion of cells to additively manufactured Ti-6Al-4V implants of after HIP, sandblasting, and polishing was evaluated in our previous study [3], indicating the vital role of surface finish on cellular response to SLMed Ti-6Al-4V samples. In this study, we explore an additional surface post-processing method, which is chemical etching. Figure 11 compares the capability of surfaces in the as-fabricated condition compared to the chemically etched surfaces for a cellular response. The metabolic activity of the seeded cells on the surface of as-fabricated and etched parts in comparison with control samples (plastic coverslips) after culturing for 1, 7 and 14 days are shown in Figure 11a. It can be observed an increase in activity in all conditions, however, the rise in biochemical processes is dependent on the post-processing applied to the SLMed samples. The as-fabricated samples display a moderate rise in metabolism when compared with the etched surfaces which showcase similar activity as the control tissue culture coverslips. These control materials are specifically developed to encourage adhesion of cells, indicating that chemical etching has a profound effect on improving the cellular affinity of the SLMed surfaces. Analysis of deposited cells on both as-fabricated and chemically etched surfaces after 1, 7 and 14 days further corroborate the higher biological response elicited by the chemical process (Figure 11b,c). Cells deposited in the as-fabricated sample are limited in number with most retaining a spherical shape during the first 24 h of deposition. The lack of spreading indicates that cells are kept in their sedimentation stage, suggesting minimal surface adhesion [32] which results in lower surface spreading. Seeded cells on the as-fabricated surface shown flattening after seven days with limited surface coverage even after 14 days of culture. In contrast, the biological response of SAOS-2 cells deposited on the etched surface is more favourable, with a high degree of flattering and coverage during the first 24 h. This results in a high degree of confluence in seven days and complete coverage after 14 days, revealing the higher biological performance of the etched sample. Studies of titanium surfaces developed by Wu et al. have shown that high roughness can limit cell growth space forcing deposited cells into their sedimentation stage [33]. The poor surface finish obtained during SLM is caused by the presence of partially unmelted particles (Figure 11b). This offers limited space for cells to adhere, leading to poor initial attachment and subsequent proliferation [34] which results in the poor biological behaviour exhibited by the as-built sample. Thus, it can be seen that post-processing of SLMed parts through HF etching results in higher metabolic activity and proliferation, suggesting that biomedical AM parts should be preferentially etched.

## 4. Conclusions

In this study, Ti-6Al-4V coupons were manufactured using SLM in two build orientations (vertical and horizontal) and subsequently post-processed using a combination of HIP, sandblasting, polishing, and chemical etching. It was found vertically built samples showed higher yield strength (YS) and ultimate tensile strength (UTS) than the horizontal samples. It was shown that using HIP resulted in reducing the residual porosity within the SLMed builds as well as, transforming the martensitic needle-like α′ structure in the as-fabricated condition into the α + β structure, which resulted in an improvement of the ductility. The superior ductility with elongation of ~22% was obtained in this study for the samples built vertically with post-processing of HIP and polishing. Furthermore, it was found that etching was the most effective post-processing treatment to improve the fatigue performance, achieving the highest run-out strength of 450 MPa, followed by the HIP + PL parts that were reported previously to be the best condition for fatigue performance, but with reduced capability for cell attachment. Most importantly, it was found that chemical etching could enhance the cellular affinity of the surface while it holds high fatigue performance which is very important when tissue integration is needed at the implantation site. 

## Figures and Tables

**Table 1:** Comparison of the tensile properties of SLM Ti-6Al-4V produced in this study built in the as-fabricated (AF) and post-processed with HIP and polishing (PL) in two orientations of vertical (V) and horizontal (H) with wrought Ti-6Al-4V, wrought and annealed Ti-6Al-4V, and the ISO standard available in the literature.

Alloy	Yield Strength (MPa)	UTS (MPa)	Elongation (%)
AF-V	820.1 ± 16.5	1006.7 ± 6.3	8.7 ± 0.8
AF-H	760.9 ± 22.3	961.3 ± 50.2	4.7 ± 0.5
HIP-PL-V	864.9 ± 3.1	936 ± 3.6	21.7 ± 2.3
HIP-PL-H	866.2 ± 49.7	937.9 ± 43.3	14.1 ± 1.5
Wrought Ti-6Al-4V [24]	962	1008	19
Wrought and annealed Ti-6Al-4V [25]	790 ± 20	870 ± 10	18.1 ± 0.8
Standard ISO 5832-3 for implants for surgery [25]	>780	>860	>10
